# Numerical study on the prediction of oil recovery rates in unconventional reservoirs at high temperatures using ecologically friendly hybrid nanofluids

**DOI:** 10.1016/j.heliyon.2024.e41512

**Published:** 2024-12-26

**Authors:** Mudasar Zafar, Hamzah Sakidin, Abida Hussain, Farman Ullah, Mikhail Sheremet, Iskandar Dzulkarnain, Roslinda Nazar, Abdullah Al-Yaari, Liaqat Ali

**Affiliations:** a School of Mathematics, Actuarial and Quantative Studies (SOMAQS), Asia Pacific University of Technology & Innovation (APU), Bukit Jalil, 57000, Kuala Lumpur, Malaysia; b Department of Fundamental and Applied Sciences, Universiti Teknologi PETRONAS, Bandar, Seri Iskandar, 32610, Perak, Malaysia; c Department of Physics, University of Science and Technology, Khyber Pakhtunkhwa, Bannu, 28100, Pakistan; d Laboratory on Convective Heat and Mass Transfer, Tomsk State University, 634050, Tomsk, Russia; e Department of Petroleum Engineering, Universiti Teknologi PETRONAS, Bandar, Seri Iskandar, 32610, Perak, Malaysia; f Department of Mathematical Sciences, Faculty of Science & Technology, Universiti Kebangsaan Malaysia, 43600, UKM, Bangi, Selangor, Malaysia; g School of Sciences, Xi'an Technological University, Xi'an, 710021, China

**Keywords:** Reservoir geometry, Green nanoparticles, Finite volume analysis, Mathematical modelling, Unconventional reservoir

## Abstract

Although oil extraction is indispensable for meeting worldwide energy demands and ensuring industrial sustainability, various hazards are observed. Therefore, this study examined the chemical oil recovery-related environmental consequences concerning water, soil, ecosystem, and human health damages. A numerical analysis explored the mathematical model for oil extraction from unconventional sources by utilising 3D porous prism geometries under high-temperature conditions. This unique methodology utilised environmentally friendly TiO_2_-SiO_2_ hybrid nanoparticles, which were not previously investigated. The optimal conditions for oil extraction were then determined by simulations performed at 100 °C, 150 °C, and 200 °C for 2 h, 4 h, 8 h, and 12 h. This study also explored the optimisation of recovery rates by analysing several variables using ANSYS Fluent software, such as flow rate, porosity, and volume fraction. Consequently, these green TiO_2_-SiO_2_ nanoparticles presented an oil recovery rate that was 28 % and 6 % higher than water-flooding and conventional monofluid injection techniques, respectively. This outcome suggested that these TiO_2_-SiO_2_ nanoparticles could enhance efficiency and minimise environmental damage.

## 1 Introduction

Given the rising global demand for energy and the inherent significance of oil in several sectors, oil extraction outputs from current reservoirs have been steadily increasing [1]. The international daily consumption of oil (or oil-based products) amounts to approximately 100 million barrels, rendering it an indispensable resource for enterprises, transportation, and daily activities. Hence, enhancing oil recovery addresses this demand while guaranteeing a consistent energy supply and a resilient economy [2]. Oil reservoirs can also be defined into two primary categories within the petroleum industry: (i) conventional and (ii) unconventional oil reservoirs [3]. Nevertheless, oil recovery from unconventional reservoirs is more difficult following its increased volume and intricate formation [4].

Oil extraction is generally performed in three stages: (i) primary, (ii) secondary, and (iii) enhanced oil recovery (EOR) stages [5]. Despite only 5 %–10 % of oil being successfully extracted from the reservoirs within the primary recovery stage, 40 % of the initial oil deposited in the reservoir can be retrieved in the secondary (or intermediate) recovery stage. Finally, the EOR stage employs cutting-edge techniques and technology to yield the highest possible oil recovery amounts. This stage usually involves various recovery techniques, which can be categorised into three primary types: (i) chemical flooding, (ii) thermal recovery, and (iii) miscible recovery [6].

A significant correlation has been denoted between nanotechnology and oil recovery capacity within the oil industry. These nanoparticles have a substantial capacity to optimise oil extraction from unconventional reservoirs due to their exceptional characteristics. Numerous studies have also utilised various nanoparticles to enhance oil recovery, yielding favourable outcomes. Nonetheless, insufficient studies on green nanoparticles in stimulating oil recovery in unconventional reservoirs have been observed [6,7]. Considering that unconventional reservoir present potential unexplored resources, these reservoirs necessitate deeper examinations. These studies can then harness the capabilities of these reservoirs through research and technological advancements to improve the worldwide energy supply and lower the reliance on traditional reserves.

A deeper understanding of the geology and extraction processes of unconventional reservoirs is crucial for maximising recovery programmes and satisfying future energy requirements. One example involves flow geometry, which is critical to oil recovery within unconventional reservoirs due to its significant influence on fluid movement mechanics and oil extraction effectiveness. Any variations in flow geometries (radial, linear, or complicated patterns) can then affect the fluid dynamics inside the reservoir rock, including flow rates, pressure distribution, and sweep efficiency [[8], [9], [10], [11], [12], [13], [14], [15]]. Even though these techniques can significantly enhance oil recovery rates, various chemicals are emitted during these processes as environmental pollutants. This process can adversely affect human health [[15], [16], [17], [18], [19], [20]].

The oil industry and researchers should devise innovative techniques and identify avenues to address various challenges for safeguarding the environment and human well-being while meeting the energy supply requirements. These ecologically sustainable techniques can decrease the need for hazardous reagents and poisonous auxiliary chemical products [21]. One notable example is green nanofluids of different nanoparticle types dispersed throughout traditional or naturally existing base fluids. These fluids indicate the necessity to implement environmentally friendly energy sources for improved efficiency of thermal management systems by reducing reliance on fossil fuels [[22], [23], [24], [25]].

**Fig. 1:**
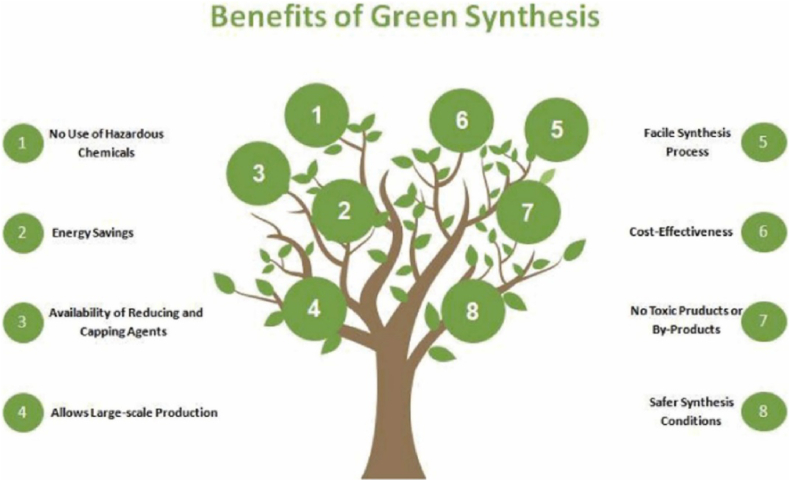
The advantages of using green technology globally [16].

Fig. 1 depicts the advantages of physical and chemical approaches for green synthesis. This procedure usually produces nanoparticles straightforwardly and undergoes quick reactions at low temperatures. Green synthesis is non-toxic and uses clean, safe, and environmentally beneficial chemicals. Furthermore, the process utilises renewable resources and is more cost-effective than current methods. The resulting green nanoparticles from this approach also reduce, cap, and stabilise contaminants by utilising phytochemicals produced from plants compared to hazardous substances. Therefore, eco-friendly nanoparticles exhibit more outstanding biocompatibility than nanoparticles produced by physical and chemical methods. Another benefit of green synthesis is energy savings, which are achieved by eliminating the need for high pressure and temperature [[24], [25], [26], [27], [28]].

Gruesbeck and Collins [29] gave an expression to evaluate the deposition of nanoparticles depending on the injected velocity which helps to give a better prediction of the loss of nanoparticles. Ju and Fan [30] revised the model to enhance the accuracy of surface deposition estimation. The model not only helps to anticipate the permeability and porosity of the reservoir, but it also forecasts the amount of oil that will be recovered after nanofluids have been injected into it.

Goldberg et al. [31]investigated how well nanoparticle transport models could predict flow in a porous medium that has been filled. The flow equation, the deposition term, remobilization, and the obstacle that induced nanoparticle transfer were utilised to classify the models developed by Salama et al. [32]. According to the author, modelling selection does not always affect nanoparticle prediction outcomes. A mathematical model of nanoparticle transport in porous media was developed by Ju and Fan [33] based on the following hypotheses: (a) the flow is one-dimensional under isothermal conditions due to the incompressibility of rock and fluids; (b) the porous medium is inconsistent; (c) Darcy's law governs the flow of water and oil in porous media, disregarding gravity; (d) nanoparticles are discretized into n-sized intervals; and (e) fluid viscosity and density are constant. The model not only helps to anticipate the permeability and porosity of the reservoir, but it also indicates the amount of oil that will be recovered after nanofluids have been injected into it. Following the injection of nanoparticles into the reservoir, Ju and Dai [33] devised equations that could be used to determine how the porosity and permeability of the reservoir would vary. The issue involved with the model is overcome using a method known as implicit pressure-explicit saturation. The computation of nanoparticle mobility in a porous medium has been properly projected using this approach. This model is an early and influential one that has been frequently referenced by researchers in later improvements to comprehend the movement of nanoparticles in porous materials. It has been used to understand the movement of nanoparticles in porous materials. Cullen et al. [34]suggested computing the degree of nanoparticle entrapment. The researchers used the Zhang et al. [35,36] two-site model to compute the loss term.

Sepehri et al. [37]employed the carbonate system model and verified its accuracy through experimental data. The model showed that changes in wettability led to an 8–10 % increase in the recovery factor when compared to the usual water flooding enhanced oil recovery (EOR) method. Accurately determining the nanoparticle loss term is crucial for accurately predicting the movement of nanoparticles in porous materials. The wettability of the core surface and the well's displacement were predicted using numerical simulations using the drag reduction model established by Chen et al. Abdelfatah [38,39] made a mathematical model for heterogeneous porous media in nanofluid injection to find the best nanoparticle physical properties for EOR uses.

El-Amin et al. [40,41]developed numerous adjustments for nanoparticle multiphase flow in porous media. Additional adjustments were made to CO2 sequestration by Ref. [42]. The equations were solved using a variant of the iterative IMPES approach that the authors tweaked to increase computer performance [42,43].

According to the preceding literature, our basic understanding of nanoparticle assisted EOR is extremely limited. We require a more in-depth theoretical understanding of how nanoparticles behave in different reservoirs and under different physical conditions. As a result, further models based on various methods for nanoparticle flow in porous media are required. Microscale mathematical models for nanoparticle flow in porous media for EOR applications are scarce in the literature. Microscale models can provide a clear knowledge of the physical interactions between nanoparticles, rocks, tiny particles, and fluid within the reservoir, as well as insight into the chemical reactions occurring within the reservoir. A study that incorporates both macro- and micro-scale models could provide a more accurate and conclusive assessment of the benefits of nanofluid-assisted EOR. Along with nanoparticles, other mechanisms, such as electromagnetic heating and magnetic fields, are used in EOR applications. As a result, it is critical to investigate the behavior of nanoparticles in the presence of magnetic fields in the reservoir between nanoparticles, oil, and water.

Previous studies neglected to explore the mathematical analysis of a 3D cavity utilising environmentally friendly nanoparticles. In this work, the unconventional reservoir plays a significant role to investigate the complicated geometrical features that influence the performance of EOR processes. Uneven reservoirs (e.g., tight oil reservoirs, shale reservoirs) are often characterized by heterogeneous porosity, low permeability, and intricate geologic formations, thereby rendering them challenging to economically produce oil through traditional conventional methods. In our research, we propose a 3D, hypothetical, prism-like vault for calculation of maximum oil production presented as sustainable hybridization nanofluid subjected to high-temperature conditions. By understanding such reservoirs, we can enhance the modeling of how such unique properties affect oil recovery and subsequently refine EOR strategies on such complex terrains and advance the oil recovery efficiency. Nevertheless, as the industry transitions toward unconventional reservoirs in order to satisfy expanding energy demands, there is a critical need to develop and validate mathematical models that are capable of modeling such complexity if greater effectiveness of EOR is to be achieved to maximize ultimate recovery from these hard-to-produce energy resources. Despite that mathematical models for EOR utilising various geometries were primarily developed by these studies, green bio-hybrid nanoparticles in a 3D hypothetical prism reservoir under high-temperature conditions were not investigated. Thus, this study established a mathematical model concerning the 3D porous geometry of green hybrid nanoparticles. The model then forecasted the oil recovery process under elevated temperatures over different time intervals.

## 2 Mathematical model

This study assessed a two-phase model to ascertain the oil recovery rate in a 3D prism geometry for an unconventional resource. The results were expressed as a systematic mathematical equation. A numerical solution to the mathematical model was also obtained using a finite volume method (FVM) solver included in the ANSYS Fluent software based on nonlinear partial differential equations.

### 2.1 Assumptions

Several assumptions are specified to develop a two-phase model and effectively predict the oil recovery rate using green nanoparticles as follows:

i. The flow of fluid is unidirectional.
ii. The Darcy law is applicable in the chosen model.
iii. The nanoparticles do not undergo any chemical reactions.
iv. The motion of the fluid flow adheres to the Newton rule.
v. The rock within the reservoir is free from contaminants.
vi. The fluid flow is incompressible.
vii. The fluid flow exhibits isothermal behaviour.

### 2.2 Geometry

**Fig. 2:**
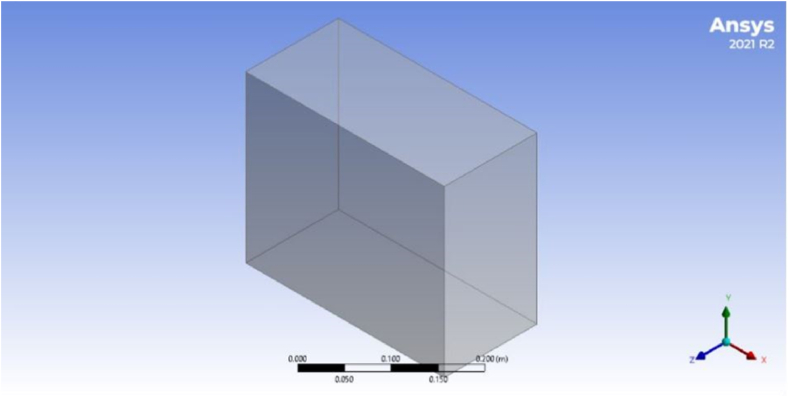
The 3D geometry of the proposed problem used to predict oil recovery rates.

An appropriate flow geometry is essential, and physical parameters should be integrated to achieve optimal outcomes in a reservoir simulation. Table 1 tabulates the parameters employed to construct the reservoir structure for simulation operations while ascertaining the physical and chemical properties of the reservoir. The green bio-hybrid TiO_2_-SiO_2_ nanoparticles were then evaluated during the flooding process to estimate the oil recovery rates at various elevated temperatures. Meanwhile, the thermal, physical, and chemical characteristics of these nanoparticles were documented in Zainon and Azmi's study [44]. Table 2 presents the physical and chemical features of the reservoir properties. Fig. 2 illustrates the geometry of the proposed problem for this study.

**Table 1:** Summary of the low oil, water, geometry, and reservoir properties [[45], [46], [47]].

Properties	Description	Quantity (SI Unit)
Core volume	The volume of the porous core inlet section of the nanofluid flow	0.049 m^3^
Inlet cross-sectional area (*A*)	Inlet cross-sectional area of the nanofluid flow	0.45 m^2^
Physical properties of the model	• Inlet fluid temperature ($T_{in}$)	300 °C
	• Initial system temperature ($T_{initial}$)	100 °C
	• Initial pressure ($P_{initial}$)	1 atm
	• Fluid outlet pressure ($P_{out}$)	1 atm
	• Initial saturation of the oil phase system ($S_{w}^{0}$)	0.20

**Table 2:** Summary of the physical and chemical properties of the reservoir [[48], [49], [50]].

Properties	Description	Quantity (SI Unit)
Light Oil properties at 300 K	• Oil density ($\rho_{o}$)	900 kg/m^3^
	• Oil heat capacity ($C_{0}$)	2000 J/Kg·K
	• Oil thermal conductivity ($k_{0}$)	0.13 W/m·K
	• Oil viscosity ($\mu_{o}$)	1.15 × 10^2^ Pa s
Water properties at 300 K	• Water density ($\rho_{w}$)	990 kg/m^3^
	• Water heat capacity ($C_{w}$)	4200 J/kg·K
	• Water thermal conductivity ($k_{w}$)	0.6 W/m·K
	• Water viscosity ($\mu_{w}$)	10^−3^ Pa s
Properties of the rock reservoir	• Rock density	2714 kg/m^3^
	• Mesh diameter ($d_{g}$)	3 μm

In the next section the mathematical model to predict oil recovery in 3D prism geometry under the influence of high temperature is explained in the form of the nonlinear Partial Differential Equations.

### 2.3 Mathematical equation

#### 2.3.1 Transport equation in porous medium

The mathematical model defined in equations 1, 2 are the nonlinear partial differential equations of water and oil phase of the nanofluid in the cavity.

Water phase:

1 $$\nabla .\left(\frac{kk_{rw}}{\gamma_{w}\mu_{w}}\nabla p_{w}\right)+q_{w}=\frac{\partial }{\partial t}\left(\epsilon \rho \frac{s_{w}}{\gamma_{w}}\;\right)$$

Oil phase:

2 $$\nabla .\left(\frac{kk_{ro}}{\gamma_{o}\mu_{o}}\nabla p_{o}\right)+q_{o}=\frac{\partial }{\partial t}\left(\epsilon \frac{s_{o}}{\gamma_{o}}\;\right)$$

where ϵ denotes the absolute permeability; $k_{rw}$ denotes the relative permeability (volume factor of the fluid); q denotes the fluid injection rate; ρ denotes the density; s denotes the saturation; $\mu_{w}$ denotes the viscosity; $p_{o}$ denotes the pressure of the fluid; and subscripts w and o denote the water and oil phases, respectively. The sum of water and oil saturations in the unconventional reservoir is also measured to be one as follows [51]

3 $$S_{o}+S_{w}=1$$

4 $$P_{o}-P_{w}=P_{c}$$

Subsequently, the μ and ρ values must be computed before solving Eqs. 1, 2) using the following equations:

5 $$\frac{1}{\mu }=s_{w\;}\frac{k_{rw}}{\mu_{rw}}+s_{oil\;}\frac{k_{r0}}{\mu_{o}}$$

6 $$\rho =s_{w\;}\rho_{w}+s_{o}\rho_{o}$$

#### 2.3.2 Transport equation for green nanoparticles

In this section the mathematical equations for the green nanoparticles are defined.

7 $$u_{w}\frac{\partial \psi }{\partial t}+\epsilon S_{w}\frac{\partial \psi }{\partial t}-\nabla \left(\epsilon S_{w}D_{ο}\nabla \psi \right)+R_{\alpha }=0$$

where u represents the fluid velocity; ψ represents the volume concentration of green nanoparticles in the water phase; $D_{ο}$ represents the dispersion coefficient; $R_{\alpha }$ represents the net loss of composition in the water phase [51]. This net loss can be calculated using an equation expressed as follows:

8 $$R_{\alpha }=\frac{\partial \varpi_{i}}{\partial t}+\frac{\partial \varpi_{i}^{*}}{\partial t}$$

where $\varpi_{i}$ defines the mass of the green nanoparticles in contact with the water phase on the pore surface per unit bulk volume of sandstone; $\varpi_{i}^{*}$ defines the mass of the green nanoparticles entrapped in the pore throat. Both terms can then be measured utilising an equation formulated as follows:

9 $$\frac{\partial \varpi_{i}}{\partial t}=K_{d}\upsilon C,\frac{\partial \varpi_{i}^{*}}{\partial t}=K_{p}\upsilon C$$

Lastly, $D_{ο}$ can be computed using an equation expressed as follows:

10 $$D_{o}=\frac{k_{rw}}{\mu_{w}}+K\;\left(\;s_{w}-1\right)\;\frac{\partial p_{c}}{\partial s_{w}}$$

#### 2.3.3 Initial and boundary conditions

The problem in this study is characterised by the initial and boundary conditions presented as follows:

11 $$When\;t=0,original\;saturation\;of\;water\;is\;zero\;i.e.,s_{w}^{0}=0$$

12 −n.ρu=0

13 −n.q=0

14 $$\rho u=\left({s_{w}\rho }_{w}+s_{o}\rho_{o}\right)U$$

15 $$-n.\frac{k_{rw}}{\mu_{w}}+K\;\left(\;s_{w}-1\right)\;\frac{\partial p_{c}}{\partial s_{w}}\nabla c_{w}=0$$

16 $$t=0,s_{w}=0.20$$

17 $$t=0,\left\{ \begin{array}{c} \psi =0\\ \;\psi =\psi_{i} \end{array}\right.$$

18 $$t=0,\left\{ \begin{array}{c} \varpi_{i}^{*}=0\\ \;\varpi_{i}=0 \end{array}\right.$$

## 3 Methodology

This study simulated the problem proposed in this study using the ANSYS Fluent software [52]. The problem-solving approach comprises three main steps as follows:

i. Step 1: A mathematical model for the 3D prism geometry was constructed to forecast the oil recovery rates in unconventional reservoirs.
ii. Step 2: The geometry and mesh were generated. Simulations were also conducted utilising various porosity, volume fraction, and mass flow rate parameters.
iii. Step 3: The accuracy of the model was verified with the experimental data documented in previous studies. An empirical study was also performed.

### 3.1 Mesh analysis

**Fig. 3:**
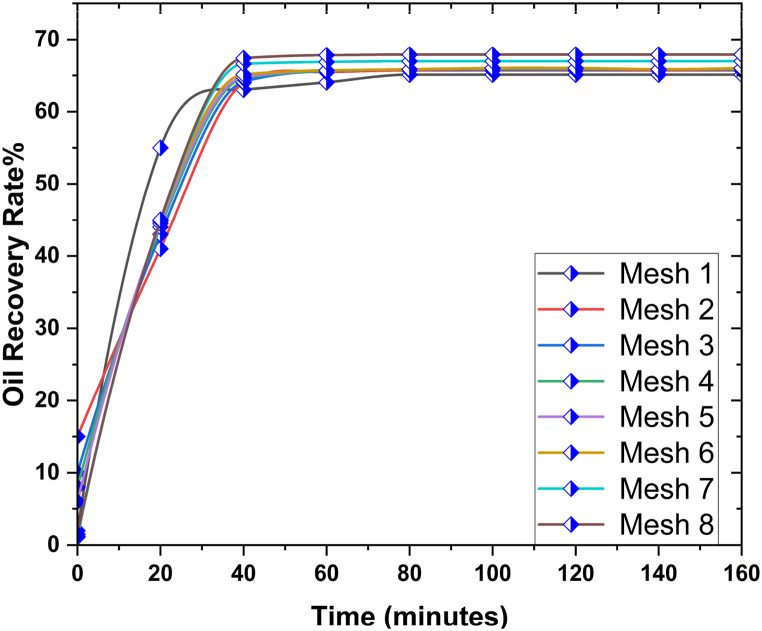
The graphical analysis of grid independency analysis for the proposed problem.

**Fig. 4:**
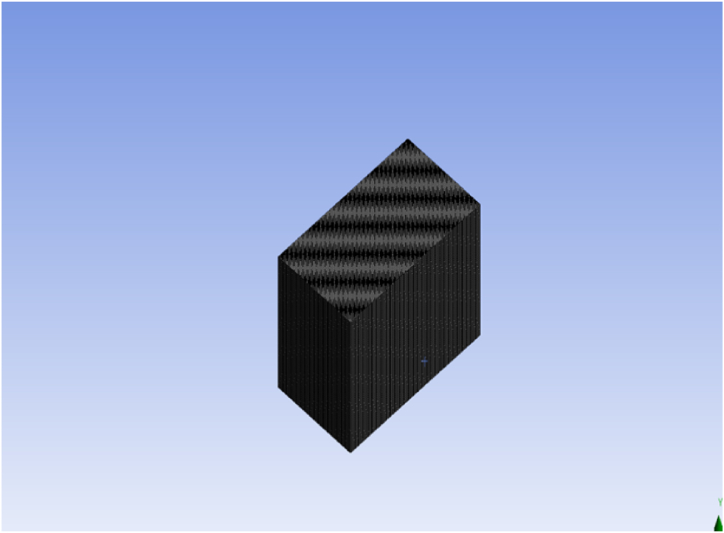
The mesh analysis of the given geometry.

A mesh analysis step was crucial to evaluate the numerical models used in simulations while assessing the quality and dependability of the outcomes. Therefore, this study employed a mesh with the maximum cell density achievable for the simulation analysis approach. The proposed model was initially solved on eight separate grids before proceeding to the validation of the oil recovery parameters across time. Fig. 3 displays the dependence implications of the grid, in which Grids 6, 7, and 8 exhibit significant similarities in several aspects. This observation suggested that the mesh size did not inherently influence the overall visual aspect of the object. Consequently, Grid 8 (325,230 nodes) was the most optimal mesh to be utilised in this study (see Fig. 4).

### 3.2 Validation

**Fig. 5:**
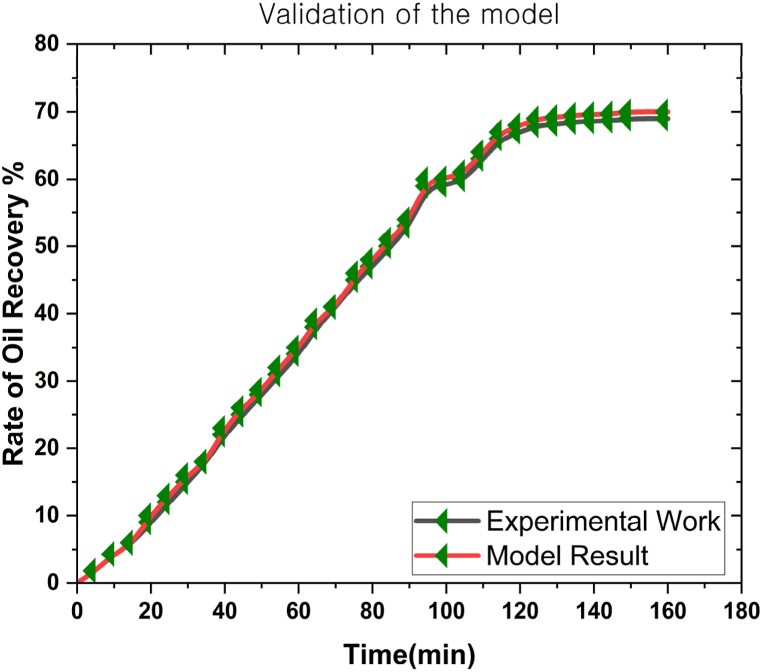
The model validation with the experimental outcomes [53].

This study aimed to quantify the oil recovery rates using green bio TiO_2_-SiO_2_ nanoparticles on a 3D prism. Thus, a comparative analysis between the model outcomes and empirical investigations was essential to verify the results. Table 3 summarises the characteristics of the experimental configuration. Fig. 5 illustrates the comparison between experimental data and model predictions. The experimental data and the model exhibited significant congruence similarities.

**Table 3:** Summary of the experimental configuration characteristics [53].

Property	Value
Length (cm)	12
Diameter(cm)	3.80–3.82
Porosity	0.23–0.25
Permeability (mD)	13.8–14.0
Reservoir temperature (^o^F)	205
Molecular weight (atomic mass units)	354
Reservoir pressure (psi)	4650

## 4 Results and discussion

This study performed various simulations over different periods (2 h, 4 h, 8 h, and 12 h) at 100^o^C, 150^o^C, and 200^o^C. An analysis was then conducted to examine how flow rate, porosity, and volume fraction variables affected the maximum oil extraction rate at different temperatures. The analytical simulations were accomplished using the ANSYS Fluent software based on the FVM to model the partial differential equation system. This study examined the porosity (0.1 ≤ φ ≤ 0.5), mass flow rate (0.01 mL/min ≤ *Q* ≤ 0.05 mL/min), and nanoparticle concentration (0.01 ≤ ψ ≤ 0.05) under gravity and high temperatures at different periods.

**Fig. 6:**
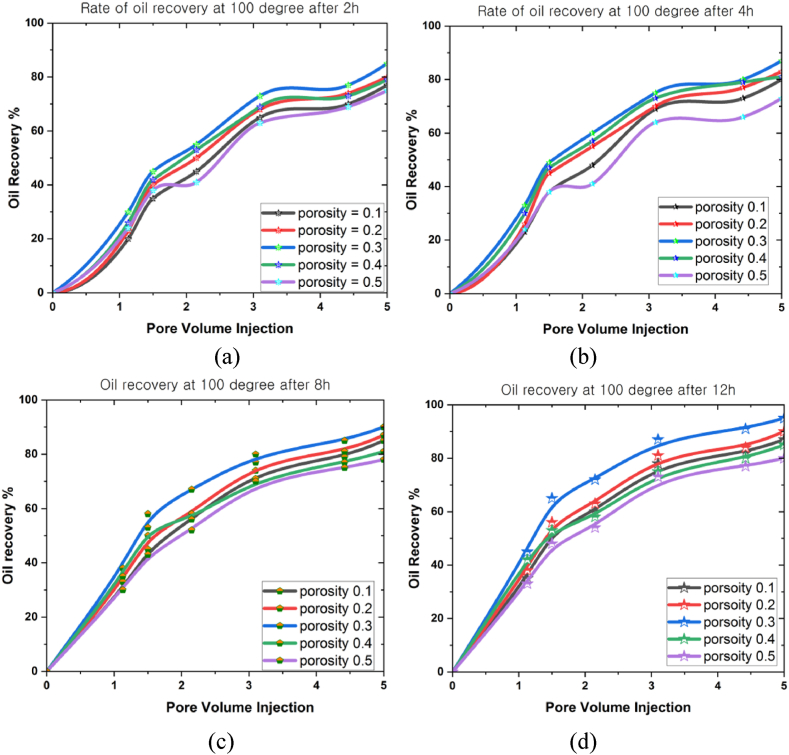
The oil recovery rates at 100^o^C under different porosities for (a) 2 h, (b) 4 h, (c) 8 h, and (d) 12 h.

Fig. 6(a)–6(d) depict the analysis performed to investigate the correlations between porosity (0.1 ≤ φ ≤ 0.5) and oil recovery rate under 100^o^C at different periods (2 h, 4 h, 8 h, and 12 h). A positive relationship between porosity and the oil recovery rate was concluded (see Fig. 6). The figures also demonstrated a proportional increase in oil recovery rates across all porosity levels when the simulation period was prolonged. An 85 % oil recovery rate was initially reported in the ultimate pore volume after 2 h. This rate peaked at 87 % after 4 h, subsequently rising to 90 % after 8 h. The oil recovery rate finally achieved 95 % after 12 h, and the porosity parameter of 0.3 in all scenarios consistently yielded the most significant oil recovery rate.

**Fig. 7:**
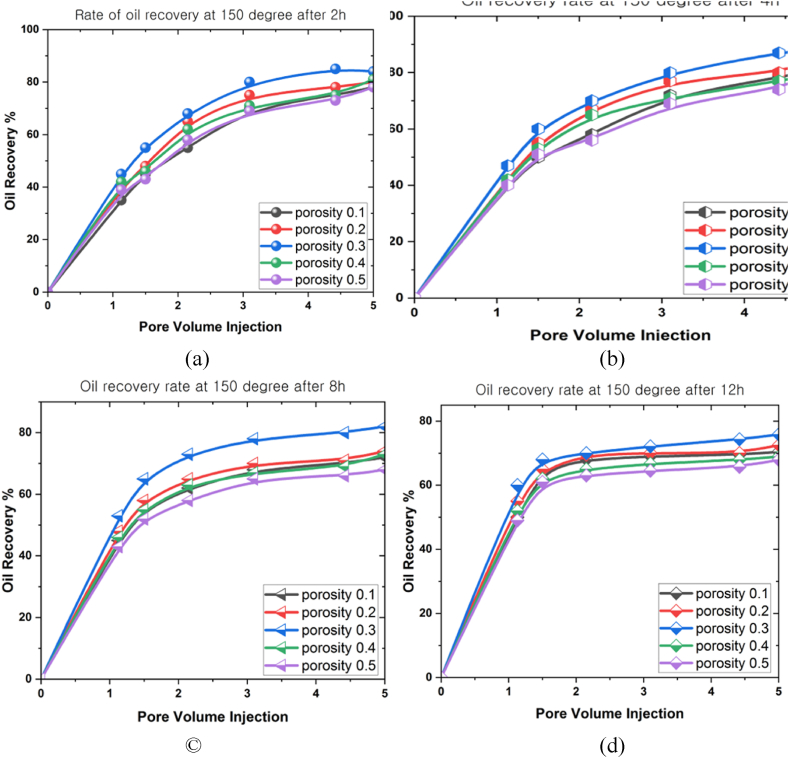
The oil recovery rates at 150^o^C under different porosities for (a) 2 h, (b) 4 h, (c) 8 h, and (d) 12 h.

Fig. 7(a)–7(d) display the analysis conducted to examine the correlations between porosity (0.1 ≤ φ ≤ 0.5) and oil recovery rate under 150^o^C at different periods (2 h, 4 h, 8 h, and 12 h). The porosity produced a substantial impact on the oil recovery rate. An 80 % oil recovery rate at the final pore volume was achieved after 2 h. Subsequently, the measured oil extraction rate achieved after 4 h was 89 %. This rate decreased to 80 % after 8 h, and the oil recovery rate finally stabilised at 75 % after 12 h. The observations indicated that the oil recovery rate at the 3D prism geometry initially increased as the temperature rose. Nonetheless, the oil recovery rate started to decline over time, suggesting that higher temperatures negatively impacted the oil recovery rate.

**Fig. 8:**
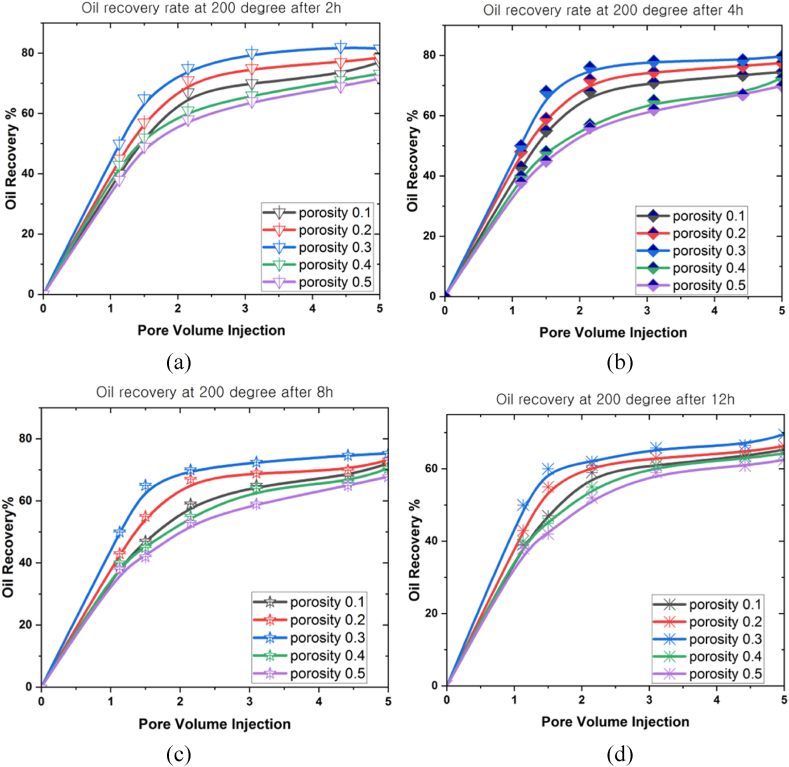
The oil recovery rates at 200^o^C under different porosities for (a) 2 h, (b) 4 h, (c) 8 h, and (d) 12 h.

Fig. 8(a)–8(d) portray the analysis performed to assess the correlations between porosity (0.1 ≤ φ ≤ 0.5) and oil recovery rate under 200^o^C at different periods (2 h, 4 h, 8 h, and 12 h). The porosity positively impacted the oil recovery rate, and the recovery rate after 2 h was 81.5 % at the final pore volume. This rate then reduced to 77 % after 4 h, which was followed by 73 % and 66 % after 8 h and 12 h, respectively. Consequently, this outcome demonstrated that the oil recovery rate initially accelerated as the temperature rose inside the 3D prism geometry. The oil recovery rate then began declining over time, indicating that higher temperatures limited the oil recovery rate.

**Fig. 9:**
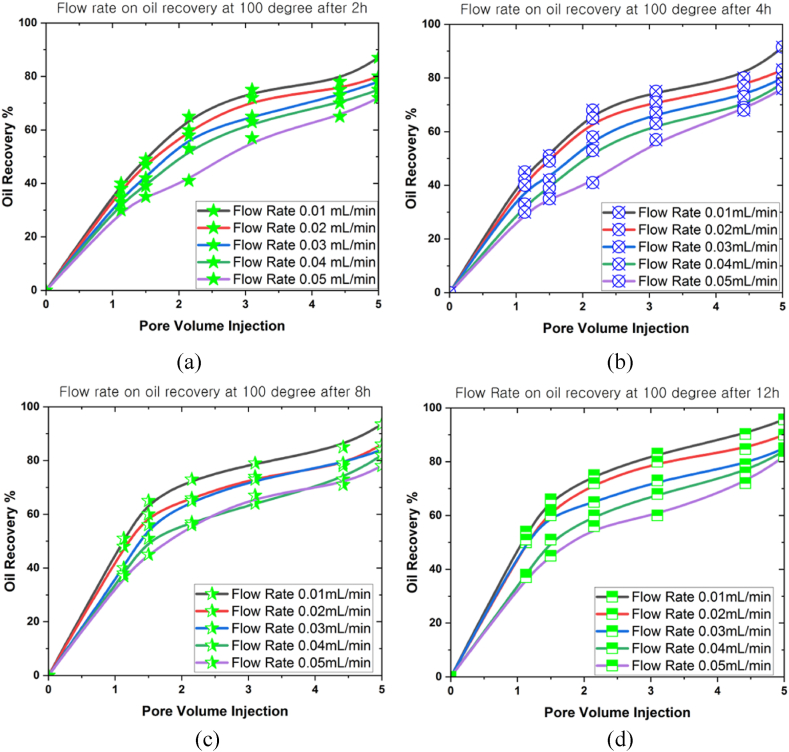
The oil recovery rates at 100^o^C under different flow rates for (a) 2 h, (b) 4 h, (c) 8 h, and (d) 12 h.

**Fig. 10:**
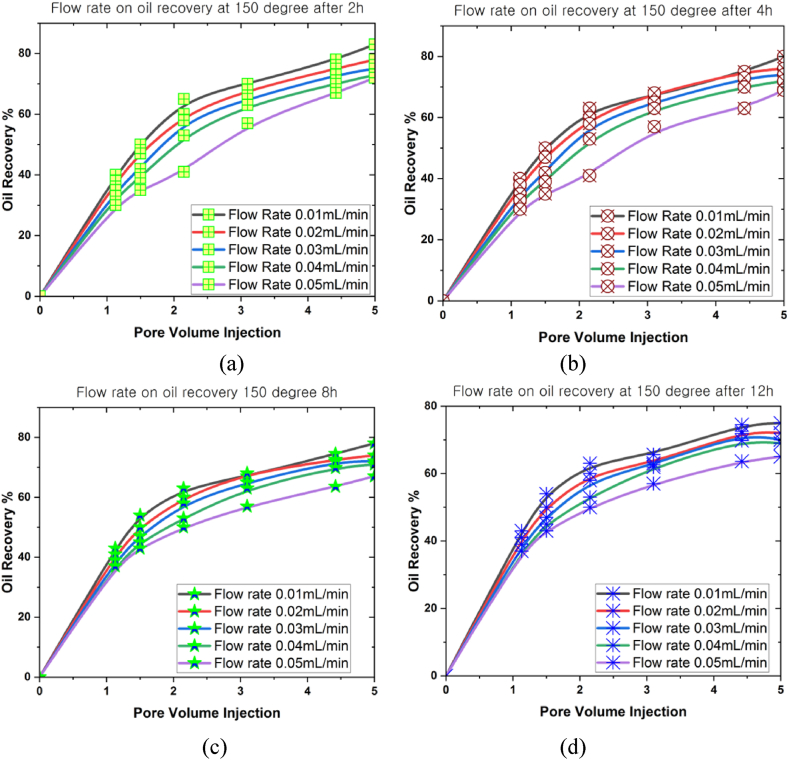
The oil recovery rates at 150^o^C under different flow rates for (a) 2 h, (b) 4 h, (c) 8 h, and (d) 12 h.

**Fig. 11:**
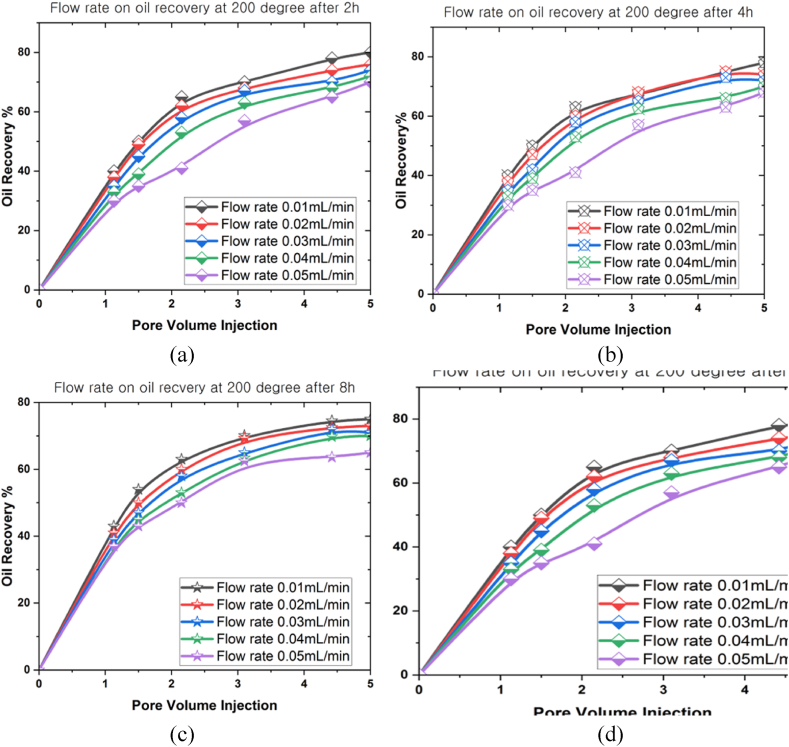
The oil recovery rates at 200^o^C under different flow rates for (a) 2 h, (b) 4 h, (c) 8 h, and (d) 12 h.

Fig. 9, Fig. 10, Fig. 11 present the correlations between flow rate (0.01 mL/min, 0.02 mL/min, 0.03 mL/min, 0.04 mL/min, and 0.05 mL/min) and oil recovery rate in a 3D prism geometry under various temperatures (100^o^C, 150^o^C and 200^o^C) at different periods (2 h, 4 h, 8 h, and 12 h). The flow rate highlighted a beneficial impact on the oil recovery rate. A negative correlation was also observed between oil recovery and flow rates for all scenarios. Notably, the oil recovery rate reached an extreme level when the simulation period grew. Meanwhile, the oil recovery rates at 100^o^C were 95.7 % (0.01 mL/min), 90 % (0.02 mL/min), 85 % (0.03 mL/min), 82 % (0.04 mL/min), and 80 % (0.05 mL/min) after 12 h. The results indicated that a higher flow rate produced lower oil recovery. Conversely, faster movement of nanoparticles inside the reservoir occurred when the flow rate was lower, promoting increased oil mobilisation. Higher temperatures inside the 3D prism also decreased the oil recovery rates, with the highest oil recovery rate achieved at 200^o^C (72 %) after 12 h.

**Fig. 12:**
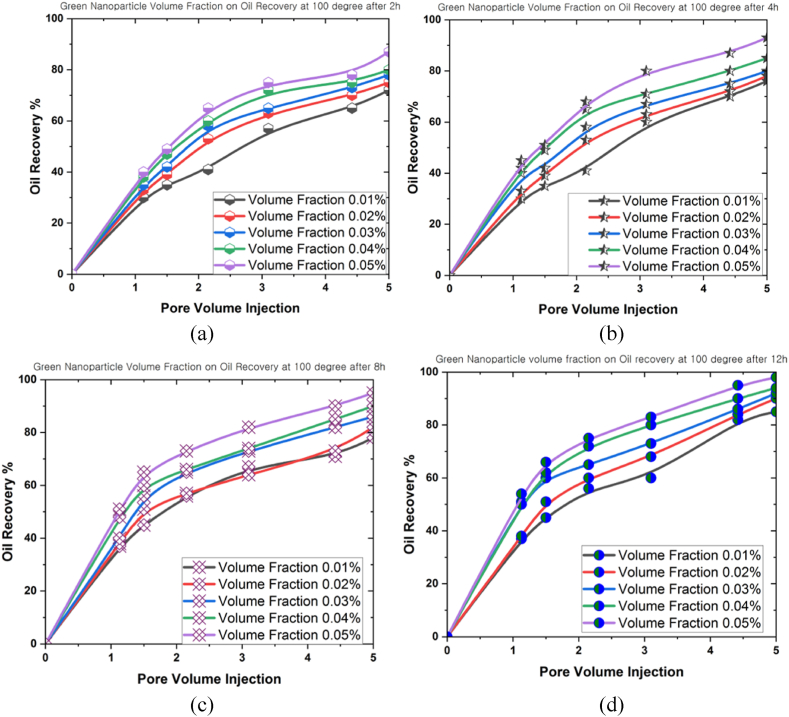
The oil recovery rates at 100^o^C under different volume fractions of green hybrid bio nanoparticles for (a) 2 h, (b) 4 h, (c) 8 h, and (d) 12 h.

**Fig. 13:**
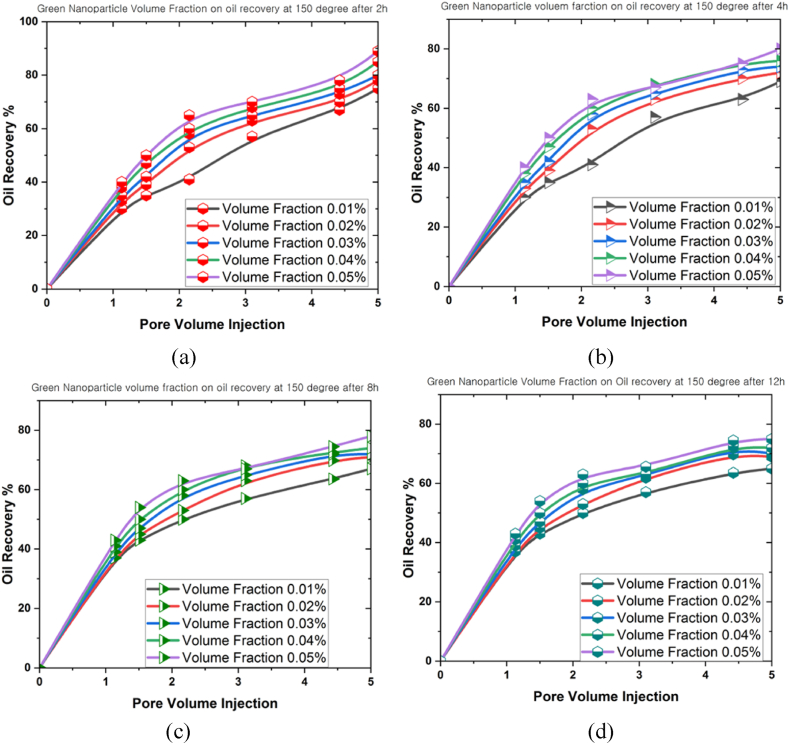
The oil recovery rates at 150^o^C under different volume fractions of green hybrid bio nanoparticles for (a) 2 h, (b) 4 h, (c) 8 h, and (d) 12 h.

**Fig. 14:**
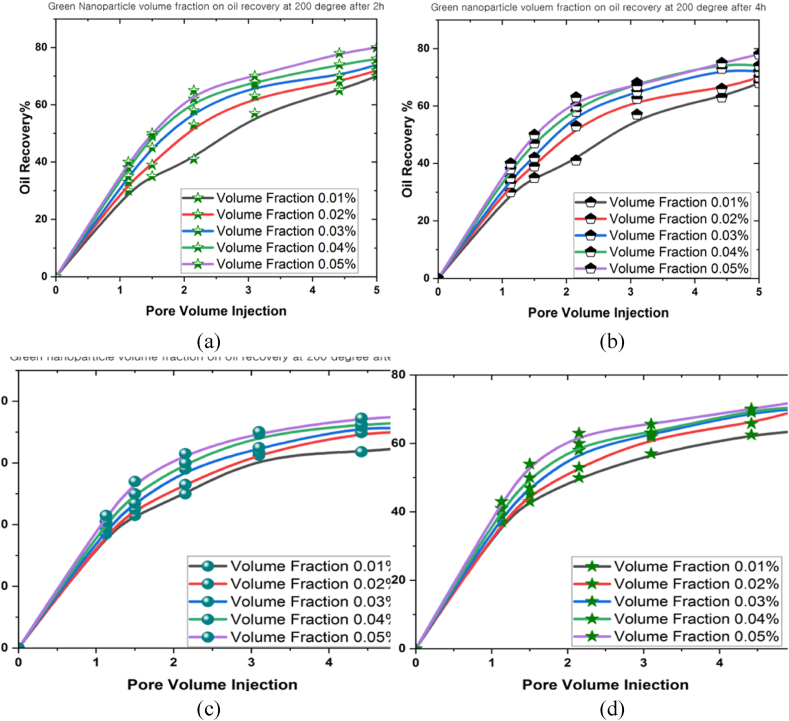
The oil recovery rates at 200^o^C under different volume fractions of green hybrid bio nanoparticles for (a) 2 h, (b) 4 h, (c) 8 h, and (d) 12 h.

Another factor contributing to the decline in oil recovery was thermal fluctuations. This phenomenon could induce either expansion or contraction of the fluids and rocks contained within the reservoir, reducing the recoverable oil volume. The modifications then possessed the capacity to affect the pressure distribution and flow patterns within the reservoir, potentially affecting the oil flow towards the extractive wells. Fig. 12, Fig. 13, Fig. 14 illustrate the influence of different volume fractions of green bio-hybrid nanoparticles (0.01 %, 0.02 %, 0.03 %, 0.04 %, and 0.05 %) under various temperatures (100^o^C, 150^o^C, and 200^o^C) for 2 h, 4 h, 8 h, and 12 h to forecast oil recovery rates in a 3D prism geometry.

The volume fraction variable revealed a beneficial influence on the oil recovery rate, and a positive correlation was denoted between volume fraction and oil recovery rate. Empirical evidence also suggested that the oil recovery rate peaked as the simulation time grew. The oil recovery rates at 100^o^C were 82 % (0.01 %), 86 % (0.02 %), 88 % (0.03 %), 90 % (0.04 %), and 97 (0.05 %) after 12 h. These results implied that the volume fraction produced a highly substantial influence on the oil recovery rate. On the contrary, the oil recovery rate decreased when the temperature inside the 3D prism increased. The measured oil recovery rate at 200^o^C after 12 h amounted to 70.5 %.

**Fig. 15:**
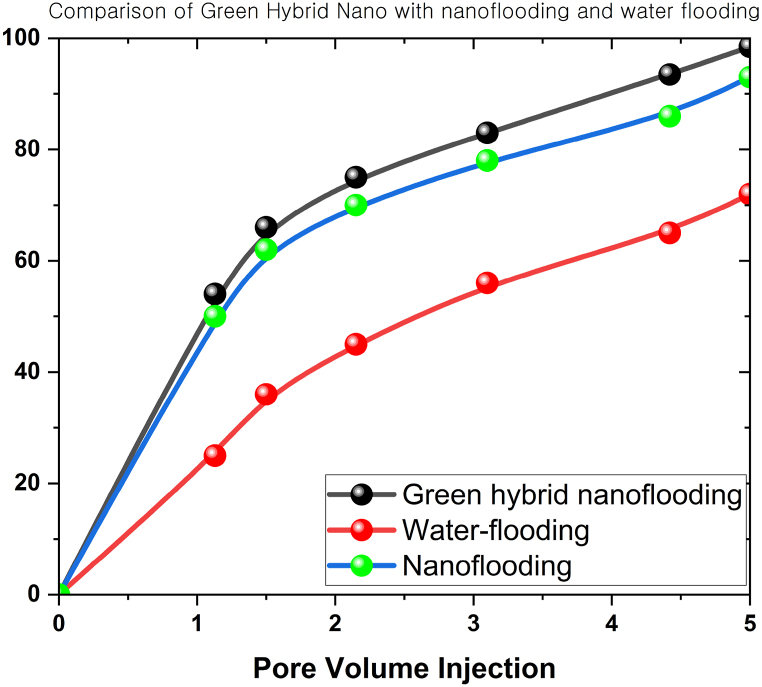
The comparison of green hybrid nanoflooding, nanoflooding, and water-flooding on oil recovery rates.

The nanoparticles could alter the fluid flow regime in the reservoir. These nanoparticles could form aggregates or clusters, inducing flow mechanism changes in the fluids within the reservoir. Therefore, this outcome demonstrated the capacity to facilitate the redirection of injected fluids (water or gases) towards regions where oil recovery was less efficient, enhancing the volume fraction and the overall recovery rate. Fig. 15 portrays the graphical comparison of hybrid green nanoflooding, nanoflooding, and water-flooding in terms of their impact on the oil recovery rate. The oil recovery rates of green hybrid nanoparticles were 27 % and 6 % greater than water-flooding and simple nanoflooding, respectively.

## 5 Conclusion

This study successfully developed a mathematical model for a 3D prism containing green bio-hybrid TiO_2_-SiO_2_ nanoparticles to forecast the oil recovery rates for unconventional oil reservoirs. Previous studies did not explore the mathematical analysis of a 3D cavity utilising environmentally friendly nanoparticles. This study conducted the numerical simulation using the ANSYS Fluent software. Several conclusions were drawn from the outcomes and findings:

i. Green nanoparticles significantly enhanced the oil recovery rate, resulting in a 27 % growth compared to water-flooding. This finding indicated that green nanoparticles held immense potential in extracting the most significant amount of oil from unconventional oil reservoirs. Moreover, these nanoparticles could mitigate environmental damage.
ii. An optimal oil recovery rate was achieved when the porosity variable was 0.3. Any increase beyond this value decreased the oil recovery rate.
iii. The temperature inside the reservoir was a crucial determinant of oil recovery enhancement. High temperatures at the initial pore volume led to a rapid increase in the oil recovery rate. Nevertheless, the oil recovery rate began declining over time.
iv. The peak oil recovery was attained at 100 °C.

## CRediT authorship contribution statement

**Mudasar Zafar:** Writing – original draft, Methodology, Investigation. **Hamzah Sakidin:** Writing – review & editing, Supervision, Investigation, Formal analysis. **Abida Hussain:** Methodology, Formal analysis, Conceptualization. **Farman Ullah:** Writing – review & editing, Methodology, Formal analysis. **Mikhail Sheremet:** Writing – review & editing, Supervision, Formal analysis. **Iskandar Dzulkarnain:** Writing – review & editing, Methodology, Funding acquisition. **Roslinda Nazar:** Writing – review & editing, Supervision, Resources, Formal analysis. **Abdullah Al-Yaari:** Writing – review & editing, Investigation, Formal analysis. **Liaqat Ali:** Validation, Formal analysis, Data curation.

## Data availability statement

The data supporting the study findings are available from the corresponding author upon reasonable request.

## Declaration of competing interest

The authors declare that they have no known competing financial interests or personal relationships that could have appeared to influence the work reported in this paper.
